# Adherence to Cancer Prevention Guidelines in 18 African Countries

**DOI:** 10.1371/journal.pone.0105209

**Published:** 2014-08-21

**Authors:** Tomi F. Akinyemiju, Jasmine A. McDonald, Jennifer Tsui, Heather Greenlee

**Affiliations:** 1 Department of Epidemiology, Columbia University, Mailman School of Public Health, New York, New York, United States of America; 2 Herbert Irving Comprehensive Cancer Center, Columbia University Medical Center, New York, New York, United States of America; Universität Bochum, Germany

## Abstract

**Background:**

Cancer rates in Africa are projected to double by 2030 due to aging and increased exposure to cancer risk factors, including modifiable risk factors. We assessed adherence to 5 modifiable cancer risk factors across 18 African countries.

**Methods:**

Data on adults 18 years and older were obtained from the 2002–2004 World Health Survey. Adherence to current World Cancer Research Fund guidelines on smoking, alcohol, body weight, physical activity, and nutrition was assessed. Adherence scores ranged from 0 (no guideline met) to 5 (all guidelines met). Determinants of adherence were assessed using multivariable linear regression adjusted for individual and country level characteristics.

**Results:**

Across all countries, adherence to the guidelines among adults was high for smoking (72%–99%) and alcohol (85%–100%), but low for body weight (1.8%–78%), physical activity (3.4%–84%) and nutrition (1.4%–61%). Overall adherence score ranged from 2.32 in Mali to 3.72 in Comoros. In multivariable models, residing in low versus high SES households was associated with reduced adherence by 0.24 and 0.21 points for men and women respectively after adjusting for age, gender, education, and marital status (p<0.001). Every % increase in GDP spent on health was associated with increased adherence by 0.03 in men and 0.09 in women (p<0.001).

**Conclusions:**

The wide variation in adherence to cancer prevention guidelines observed across countries and between population sub-groups suggests the need for targeted public health efforts to improve behaviors related to body weight, physical activity and nutrition.

## Introduction

Cancer rates in Africa are predicted to increase in the coming decades [1]. According to the International Agency for Research on Cancer, in 2008 there were approximately 681,000 new cancer cases and 512,000 cancer deaths in Africa [2]. By 2030, 1.28 million new cancer cases and 970,000 cancer deaths are expected, an 88% increase from 2008 [2]. The increase in cancer and cancer-related deaths is likely due to the transition to a decline in highly fatal infectious diseases [3], [4]. As a consequence, life expectancy rates are approaching levels never before experienced in many African countries, with the resulting challenge of managing an increasingly aging population and the associated rise in chronic diseases [2], [3], [5]. In addition, there is a rapid adoption of Western lifestyle patterns, characterized by tobacco use, low physical activity, high fat/calorie dense diet, lower parity, and shorter duration of breastfeeding [1], [4], [6]. These modifiable lifestyle risk factors have been associated with many chronic diseases, including colorectal, breast, and prostate cancer [7]–[11].

In 2007, the World Cancer Research Fund (WCRF) and the American Institute for Cancer Research (AICR) issued specific recommendations regarding smoking, alcohol use, body weight, physical activity, and diet, for the prevention of cancer using the most current and comprehensive set of scientific evidence [12]. These recommendations have been widely studied in relation to chronic diseases, and have been associated with significant reductions in cancer mortality, cancer risk, cancer aggressiveness and improved survival among adherent adults [13]–[16]. National screening programs coupled with specialized and multidisciplinary treatment have been shown to be associated with the greatest cancer mortality reduction [17]. However, developing countries in general, and African countries in particular often lack the necessary resources to develop needed secondary and tertiary prevention infrastructure. Primary prevention, focusing on modifiable risk factors, may be a more efficient cancer prevention method in these areas since it is cheaper to implement, and the benefits of adherence extends to other chronic diseases also highly prevalent in developing countries.

To our knowledge, no research study has systematically assessed adherence to these guidelines in African countries. While these guidelines were developed to provide evidence-based recommendations to individuals and communities worldwide, less is known about the application of these guidelines to African populations. Given the projected increase in cancer rates in Africa, it is important to assess adherence to these guidelines in order to identify populations to target with future interventions. The purpose of this study is to assess the level of adherence to the 2007 WCRF/AICR cancer prevention guidelines in African countries, and to examine individual and country level predictors of adherence.

## Methods

### Data Source and Analytic Samples

Data were obtained from the 2002–2004 World Health Survey (WHS) conducted in 70 countries globally. The WHS is uniquely suited for cross-country comparisons given that its high response rates and nationally representation. Details of the survey have been published previously [18]. In brief, the WHS employed a multistage cluster sampling technique in which each individual in the survey had a known, non-zero probability of being included. The cross-sectional study included adults surveyed at the household and individual levels using standardized questionnaires. As the data was de-identified and publicly available, the present analysis was exempt from IRB review.

The present analysis, conducted in 2013, focused on 18 sub-Saharan African countries: Burkina Faso, Chad, Cote D’Ivoire, Congo, Comoros, Ethiopia, Ghana, Kenya, Malawi, Mali, Mauritania, Mauritius, Namibia, Senegal, Swaziland, South Africa, Zambia, and Zimbabwe. Data were collected via in-person interviews in English or translated into local languages. Adults ages 18 years and older were included in the analysis (n = 72,571). Cancer incidence of the most common 5 sites for each country was obtained from the 2008 World Health Organization GLOBOCAN dataset [19]. Detailed descriptions of the country specific source of cancer data are listed in Table S1 in File S1. Data on country level health expenditure as a proportion of GDP was obtained from the World Development Indicators catalog [20]. Country level health expenditure represents the sum of public and private health expenditure on preventive and curative health services, family planning, nutrition activities, and emergency health services.

### Measures

#### Adherence

Five self-reported cancer related risk factors were assessed using cancer prevention guidelines established by the WCRF/AICR (12): smoking, alcohol, body weight (BMI), physical activity, and nutrition (fruit and vegetable intake). For each risk factor, a score of 1 was assigned if the guideline was completely met, 0.5 if partially met, and 0 if not met at all (Table 1). All risk factors were weighted equally and summed up to create the total adherence score ranging from 0–5. Detailed description of each item and categorization are presented in Table 1. Adherence was complete if an individual was a non-smoker; consumed ≤7 alcoholic drinks per week for women and ≤14 drinks per week for men; BMI between 18.5 and 25 kg/m^2^; engaged in ≥210 minutes of physical activity per week; and consumed ≥5 servings of fruits and vegetables per day. Similar scoring algorithms have been employed in other studies of cancer [15],[21],[22].

**Table 1:** Adherence to Cancer Prevention Guidelines Metrics, 2002–2004 World Health Survey.

Risk Factor	WHS Questions	Adherence Guideline	Adherence Score
*Physical Activity*	ƒDuring the last 7 days, on how many daysdid you do vigorous physical activities?How much time did you usually spend doingvigorous physical activities on one of those days?	≥210 minutes ofphysical activity per week	1
	‡During the last 7 days, on how many days didyou do moderate physical activities? Howmuch time did you usually spend doing moderatephysical activities on one of those days?	≥150–<210 minutes ofphysical activity per week	0.5
	*During the last 7 days, on how many days didyou walk for at least 10 minutes at a time? Howmuch time did you usually spend walkingon one of those days?	<150 minutes ofphysical activity per week	0
*Obesity (BMI)*	Your weight in Kilos? Your height in Centimeters?	≥18.5–≤25 kg/m^2^	1
		>25–<30 kg/m^2^	0.5
		<18.5 or >30 kg/m^2^	0
*Alcohol Use*	Have you ever consumed a drink that contains alcohol(such as beer, wine, etc.)? (yes/never) If Yes: Duringthe past 7 days, how many standard drinks of anyalcoholic beverage did you have each day?	≤7 drinks per week for womenand ≤14 drinks per week for men	1
		>7–≤14 drinks per week forwomen and >14–≤28drinks per week for men	0.5
		>14 drinks per week forwomen and >28 drinks perweek for men	0
*Smoking*	Do you currently smoke any tobacco products suchas cigarettes, cigars, or pipes? (daily/yes,but not daily/no, not at all)	Non-smoker	1
		Current Smoker	0
*Nutrition* *(Fruit and* *Vegetable Intake)*	How many servings of fruit do you eaton a typical day? How many servings ofvegetables do you eat on a typical day?	≥5 servings of fruits andvegetables per day	1
		>3–<5 servings of fruits andvegetables per day	0.5
		<3 servings of fruits andvegetables per day	0

ƒ Vigorous activity was defined as activities that you did for at least 10 minutes at a time that make you breathe much harder than normal (e.g. heavy lifting, digging, aerobics, or fast bicycling).

‡ Moderate activity was defined as physical activities that you did for at least 10 minutes at a time that make you breathe somewhat harder than normal (e.g. carrying light loads, bicycling at a regular pace, or doubles tennis). Participants were instructed to not include walking.

*Walking was defined as time spent walking at work and at home, walking to travel from place to place, and any other walking that a participant might do solely for recreation, sport, exercise, or leisure for at least 10 minutes at a time.

#### Socio-Demographic and Economic Variables

Data on educational attainment, marital status, region of residence, and current health status were assessed by self-report. A range of permanent household income indicators was used to create an SES index for each participant, separately for each country. Ownership of assets ranging from chairs, tables, mobile phones, refrigerators, and TVs were assessed in addition to country specific items. Principal components analysis was used to create a composite SES score at the household level, and each individual was assigned the SES score of their household of residence. This method provides a more accurate representation of SES in low-income areas compared with measures of actual income and education [23]–[26].

#### Country-Level Variable

The country’s total health expenditure as a proportion of its GDP in 2005 was used as a measure of a country’s investment in the health system including preventive and curative healthcare services, family planning, nutrition, and emergency aid services. Proportion of GDP spent on health has been used in several previous studies and showed significant associations with cancer outcomes such as screening rates [27], [28].

### Statistical Analysis

Analyses were based on survey statistical methods that accounted for complex sampling design in SAS (version 9·3, SAS Institute). Descriptive statistics were conducted to assess the percentage of adults meeting each of the adherence guidelines in each country. Average adherence score was calculated separately for each country, by gender, socio-economic status, rural/urban residence, overall health, and country health expenditure. Multivariable regression analysis was conducted to assess predictors of overall adherence to guidelines using adherence score as a continuous outcome (1-point increment). Three sequential models were fit to assess the relationship between study variables and adherence score; individual variables; household socio-economic status; and regional and country level variables.

## Results

### Sample population

Sample size for the 18 countries ranged from 1,835 in Comoros to 5,541 in Malawi. The 2008 age-adjusted incidence rates per 100,000 for the Africa region are presented in Table S1 in File S1, and were as follows: female breast, 25.2; cervix, 28; colorectal, 5.9; liver, 8.3; and prostate, 17.5. Breast cancer was most common in Mauritius (42.6 cases/100,000); prostate (59.7 cases/100,000) and colorectal (14.5 cases/100,000) cancers were most common in South Africa; cervical cancer was most common in Zambia (52.8 cases/100,000); and liver cancer was most common in Ghana (17.4 cases/100,000). In contrast, Swaziland had the lowest rates of breast (9.9 cases/100,000) and colorectal (2.2 cases/100,000) cancers, and Mauritius had the lowest rates of cervical cancer (12.9 cases/100,000).

**Figure 1:**
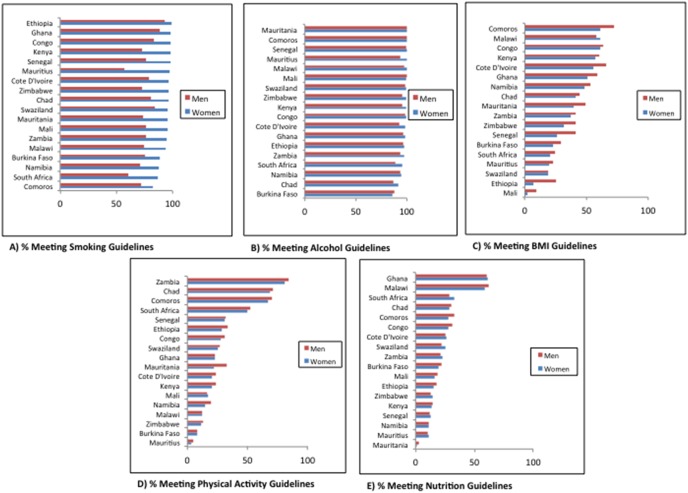
Adherence to smoking, alcohol, BMI, physical activity and nutrition guidelines, 2002–2004 WHS.

**Figure 2:**
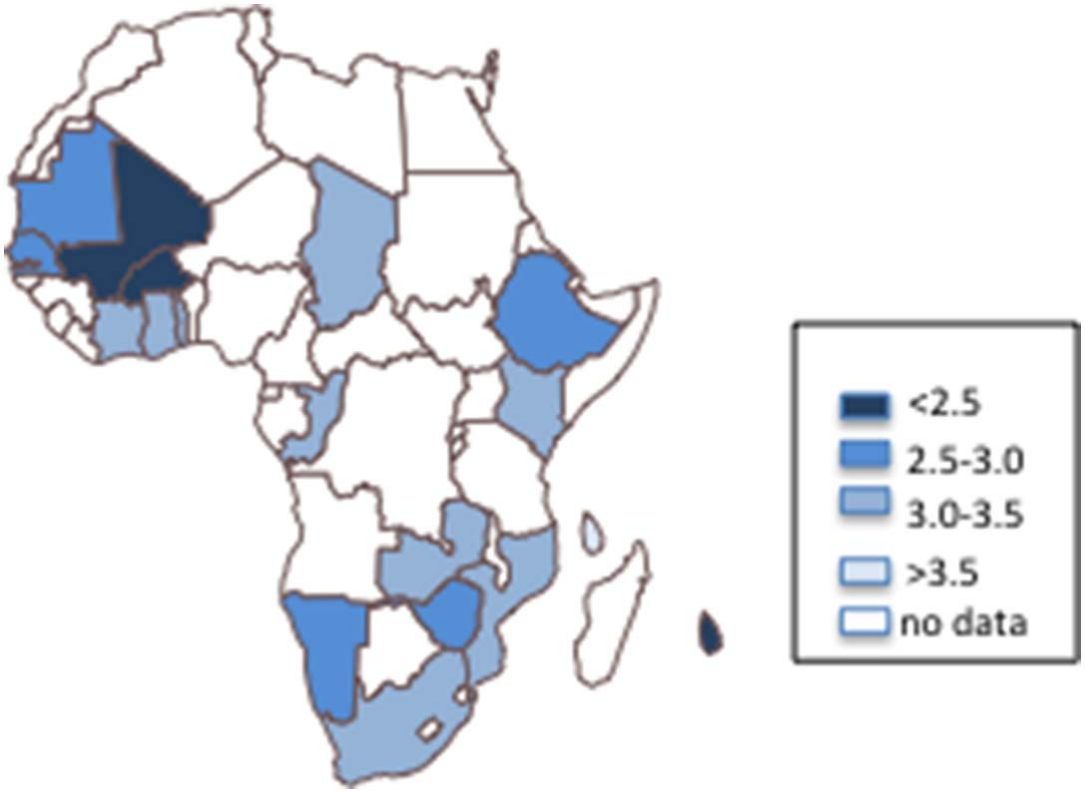
WCRF adherence score categories for African countries represented in the 2002–2004 WHS.

The distribution of individual characteristics and adherence components are presented in Table 2. Less than 5% of study participants reported any college education. Mean overall adherence score was 2.82 for women and 2.72 for men. The proportion of men and women in each country meeting each of the cancer prevention guidelines are presented in Figure 1 (detailed figures presented in Table S2 in File S1). Overall adherence score by country are presented in Figure 2.

**Table 2:** Distribution of Socio-Demographic and Adherence Measures among World Health Survey Participants in sub-Saharan Africa.

	Women (n = 36,599)		Men (n = 31,195)	
	n	% (Std. Error)	n	% (Std. Error)
**Socio-Demographic Characteristics**				
Age Category				
18–29	13,748	20.0 (0.36)	11,052	20.0 (0.43)
30–49	14,307	19.6 (0.31)	12,680	19.23 (0.40)
50+	8,544	10.9 (0.30)	7463	10.2 (0.23)
Education				
< = Primary School	30,283	37.8 (0.5)	23,174	32.8 (0.6)
Secondary school	5,319	10.7 (0.4)	6,341	13.8 (0.5)
College Plus	921	2.0 (0.3)	1,607	2.7 (0.2)
Marital Status				
Married/Cohabiting	22,032	29.5 (0.38)	19,716	29.6 (0.56)
Separated/Divorced	2,926	3.6 (0.21)	1,084	1.23 (0.09)
Widowed	4,937	5.5 (0.19)	745	0.9 (0.06)
Never Married	6,556	12.0 (0.35)	9529	17.7 (0.46)
Setting				
Rural	23,325	34.3 (0.59)	19,357	33.1 (0.70)
Urban	13,271	16.2 (0.62)	11,838	16.4 (0.62)
Overall Health				
Good	22,685	33.9 (0.43)	21,431	36.3 (0.51)
Moderate	9,707	12.4 (0.3)	7,010	10.4 (0.31)
Poor	4,065	4.2 (0.17)	2,621	2.8 (0.13)
Household SES				
Low	9,525	15.4 (0.47)	7,179	13.8 (0.57)
Middle	8,894	17.3 (0.44)	7,805	17.3 (0.41)
High	8,392	17.5 (0.61)	7,998	18.6 (0.57)
**Adherence Measures**				
Smoking				
Never	33,591	48.3 (0.51)	22,588	38.7 (0.55)
Current	1,829	2.4 (0.16)	7,291	10.6 (0.36)
Alcohol				
< = 7 Drinks/week	35,639	49.1 (0.50)	28,337	44.4 (0.48)
7–14 Drinks/week	433	0.8 (0.07)	1259	2.1 (0.15)
>14 Drinks/week	527	0.7 (0.07)	1599	2.9 (0.20)
BMI				
18.5–25.0 kg/m	18,266	30.7 (0.5)	14,309	25.8 (0.61)
25.0–30.0 kg/m^2^	5,025	5.1 (0.21)	4,044	5.3 (0.25)
<18.5 or >30 kg/m^2^	13,308	14.72 (0.38)	12,842	18.4 (0.39)
Physical Activity				
<150 minutes/wk	32,897	41.8 (0.53)	27,300	40.2 (0.59)
150–210 minutes/wk	72	0.5 (0.07)	64	0.35 (0.06)
>210 minutes/wk	3630	8.3 (0.42)	3831	8.8 (0.39)
Fruits and Vegetables				
<3 servings/day	19,035	25.4 (0.55)	16,633	25.9 (0.65)
3–5 servings/day	8,972	12.8 (0.36)	7,205	11.7 (0.33)
> = 5 servings/day	8,592	12.3 (0.39)	7,357	11.8 (0.42)
Adherence Score				
0–2	11,315	19.4 (0.54)	12,030	21.5 (0.67)
3–4	18,518	28.4 (0.57)	14,172	26.7 (0.57)
> = 4	1,087	2.1 (0.22)	1,023	1.8 (0.14)
Mean (Std. Error of Mean)	2.82 (0.02)		2.72 (0.02)	

### Smoking and alcohol

Adherence to smoking and alcohol guidelines were generally high for most of the countries (Figures 1). Adherence to smoking guidelines ranged from 83% in Comoros to 99% in Ethiopia for women, and ranged from 61% in South Africa to 93% in Ethiopia for men. Adherence to alcohol guidelines ranged from 86% in Burkina Faso to 100% in Comoros and Mauritania for women, and ranged from 87% in Burkina Faso to 100% in Mauritania for men.

### BMI, physical activity, nutrition

Adherence to BMI, physical activity, and nutrition were less encouraging (Figures 1). For women, adherence to BMI guidelines ranged from under 2% in Mali to 62% in Comoros and for men ranged from 9% in Mali to 73% in Comoros. Adherence to physical activity guidelines for women ranged from under 3% in Mauritius to 81% in Zambia, and for men ranged from under 5% in Mauritius to 84% in Zambia. Adherence to nutrition guidelines was also low with adherence for women ranging from under 2% in Mauritania to 61% in Ghana, and for men ranging from 2.5% in Mauritania to 60% in Ghana.

### Country-specific adherence scores

Table 3 presents the overall country-specific adherence scores. Comoros had the highest adherence to cancer prevention guidelines, (3.72 out of 5). The lowest score was observed in Mali (2.32). Adherence appeared to be higher in younger age groups (2.89 in ages 18–29 versus 2.62 in ages 50+); in women compared with men (2.82 versus 2.72); in highest SES households compared with the lowest (2.99 versus 2.69); in urban areas compared with rural areas (2.89 versus 2.71), and among people with good overall health status compared with poor overall health (2.81 versus 2.69). However, adherence trends were different for some countries. In Comoros and Ethiopia, adherence was higher among men compared with women; and in Cote d’Ivoire and Ethiopia, adherence was higher in residents of lowest SES household compared with the highest.

**Table 3:** Adherence Score (Range 0–5) by Country and Selected Socio-Demographic Characteristics, World Health Survey.

		Age Group			Gender		Household SES			Overall health			Regional Setting	
Country	Total	18–29	30–49	50+	Female	Male	Low	Middle	High	Poor	Mod	Good	Rural	Urban
**Burkina Faso**	2.37	2.57	2.38	2.02	2.38	2.37	2.27	2.35	2.56	2.10	2.30	2.42	2.33	2.60
**Chad**	3.38	3.51	3.34	3.22	3.44	3.32	3.31	3.41	3.46	3.05	3.42	3.44	3.37	3.44
**Cote D’Ivoire**	3.16	3.19	3.15	3.06	3.21	3.12	3.20	3.20	3.13	3.05	3.10	3.20	3.28	3.10
**Congo (Republic)**	3.46	3.54	3.44	3.28	3.46	3.45	3.46	3.42	3.66	3.17	3.42	3.52	3.42	3.46
**Comoros**	3.72	3.87	3.81	3.47	3.69	3.76	3.62	3.93	3.65	3.39	3.70	3.82	3.73	3.72
**Ethiopia**	2.66	2.82	2.62	2.41	2.57	2.75	3.36	2.72	2.92	2.39	2.44	2.74	2.63	2.88
**Ghana**	3.50	3.57	3.49	3.43	3.53	3.47	3.14	3.20	3.10	3.28	2.44	3.54	3.60	3.38
**Kenya**	3.06	3.18	2.96	2.93	3.15	2.95	3.00	3.06	3.07	2.93	2.95	3.11	3.08	2.93
**Mali**	2.32	2.34	2.32	2.31	2.36	2.30	2.18	2.36	2.39	2.17	2.29	2.34	2.29	2.41
**Mauritania**	2.72	2.74	2.70	2.71	2.73	2.70	2.73	2.80	2.69	2.62	2.76	2.71	2.78	2.66
**Mauritius**	2.39	2.49	2.38	2.31	2.59	2.19	2.22	2.37	2.55	2.28	2.39	2.41	2.38	2.40
**Malawi**	3.42	3.53	3.40	3.18	3.52	3.29	3.35	3.46	3.46	3.36	3.34	3.44	3.41	3.44
**Namibia**	2.79	2.93	2.77	2.55	2.81	2.76	2.65	2.81	2.87	2.65	2.69	2.83	2.74	2.87
**Senegal**	2.80	2.83	2.83	2.72	2.82	2.79	2.49	2.86	2.90	2.77	2.79	2.81	2.83	2.77
**Swaziland**	3.19	3.29	3.15	3.10	3.21	3.16	3.14	3.17	3.23	3.15	3.11	3.28	3.20	3.13
**South Africa**	3.01	3.06	2.87	2.76	2.97	2.88	2.69	2.90	2.99	2.72	2.85	2.97	2.88	3.02
**Zambia**	3.50	3.65	3.46	3.28	3.10	3.41	3.56	3.53	3.03	3.22	3.50	3.53	3.58	3.37
**Zimbabwe**	2.75	2.93	2.72	2.46	2.70	2.68	2.74	2.79	2.69	2.48	2.79	2.79	2.71	2.81
**Total**		2.89	2.72	2.62	2.82	2.72	2.69	2.90	2.99	2.69	2.59	2.81	2.71	2.89

### Predictors of adherence

Table 4 shows the multivariable models with socio-demographic and country level predictors of adherence score stratified by gender. Age, education, and overall health status significantly predicted adherence scores for both genders. Male and female participants ages 18–29 scored 0.20 and 0.18 points higher respectively, compared with participants ages 50 years and older. Participants with less than a primary school education scored on average 0.34 and 0.35 points lower for men and women respectively, compared with participants with some post-secondary or college education. Marital status was not associated with adherence for women, but for men, being separated or divorced was associated with a lower adherence score (β = 0.24). For both genders, being in poor or moderate overall health was associated with a significant reduction in adherence score compared with participants in good overall health, although the reduction was larger for men (β = –0.23) compared with women (β = –0.19).

**Table 4:** Socio-Demographics, Household and Country Level Predictors of Adherence to Cancer Prevention Guidelines¶.

	Men			Women		
	Model 1- Demographics	Model 2-Demographicsand SES	Model 3-Demographics, SESand Country Fixed Effects	Model 1- Demographics	Model 2-Demographicsand SES	Model 3-Demographics,SES andCountry Fixed Effects
Age						
18–29	0.22(0.14, 0.28)	0.19(0.18, 0.21)	0.27(0.24, 0.28)	0.18(0.10, 0.25)	0.25(0.24, 0.26)	0.33(0.32, 0.35)
30–40	0.01(−0.05, 0.06)	0.03(0.02, 0.05)	0.07(0.05, 0.08)	0.08(0.01,0.14)	0.15(0.14, 0.16)	0.20(0.18, 0.21)
50+	Ref	Ref	Ref	Ref	Ref	Ref
Education						
< = Primary school	−0.34(–0.45, –0.23)	−0.26(–0.29, –0.23)	–0.36(–0.39, –0.33)	–0.35(–0.6, –0.1)	–0.01(–0.04, 0.02)	–0.13(–0.16, –0.10)
Secondary school	−0.10(−0.21, 0.02)	0.002(−0.03, 0.04)	–0.09(–0.13, –0.06)	–0.27(–0.5, –0.1)	0.02(–0.01, 0.05)	–0.10(–0.13, –0.07)
College plus	Ref	Ref	Ref	Ref	Ref	Ref
Marital Status						
Married/Cohabiting	−0.01(−0.07, 0.06)	−0.05(−0.06, –0.04)	–0.03(–0.05, –0.02)	0.01(–0.05, 0.1)	0.01(–0.01, 0.01)	–0.01(–0.00, 0.02)
Separated/Divorced	−0.24(−0.37, –0.12)	−0.29(−0.32, –0.26)	–0.22(–0.26, –0.18)	0.04(–0.1, 0.17)	–0.01(–0.03, 0.01)	0.06(0.04, 0.09)
Widowed	−0.07(−0.22, 0.07)	0.01(−0.01, 0.04)	0.08(0.05, 0.12)	–0.04(–0.1, 0.03)	–0.02(–0.04, 0.01)	0.04(0.02, 0.06)
Never married	Ref	Ref	Ref	Ref	Ref	Ref
Overall Health						
Poor	−0.23(−0.33, –0.12)	−0.21(−0.24, –0.18)	–0.14(–0.17, –0.10)	–0.19(–0.3, –0.1)	–0.15(–0.17, –0.1)	–0.06(–0.09, –0.03)
Moderate	−0.10(−0.15, –0.05)	−0.12(−0.13, –0.10)	–0.09(–0.11, –0.07)	–0.08(–0.1, –0.1)	–0.09(–0.09, –0.1)	–0.05(–0.07, –0.04)
Good	Ref	Ref	Ref	Ref	Ref	Ref
Household SES						
Low		−0.22(−0.23, –0.20)	–0.24(–0.26, –0.22)		–0.18(–0.19, –0.2)	–0.21(–0.22, –0.18)
Middle		−0.03(−0.04, –0.02)	–0.04(–0.05, –0.02)		–0.04(–0.05, –0.1)	–0.05(–0.06, –0.04)
High		Ref	Ref		Ref	Ref
Setting						
Rural			0.01(–0.02, 0.03)			–0.05(–0.07, –0.03)
Urban			Ref			Ref
2005% GDP Spent on Health			0.03(0.03, 0.04)			0.09(0.08, 0.09)

¶ Model 1 adjusted for age, education, marital status, overall health and country. Model 2 adjusted for age, education, marital status, overall health, household SES and country. Model 3 adjusted for age, education, marital status, overall health, regional setting and country level % of GDP spent on health.

Upon adjusting for household SES, most of the demographic variables remained statistically significant, although slightly attenuated. Household SES was a strong, significant predictor of adherence for both genders. Men and women in low SES households scored 0.22 and 0.18 points lower respectively, compared with participants in high SES households. Including the regional setting and country level % of GDP spent on health in model 3 did not attenuate any of the previous associations except for overall health. There was no significant association between rural/urban settings and adherence score for men, although residing in rural regions was associated with a statistically significant 0.05 point reduction in adherence score among women. For every 1% increase in GDP spent on health, adherence score increased by a statistically significant 0.09 points on average for women, and by 0.03 points for men.

**Figure 3:**
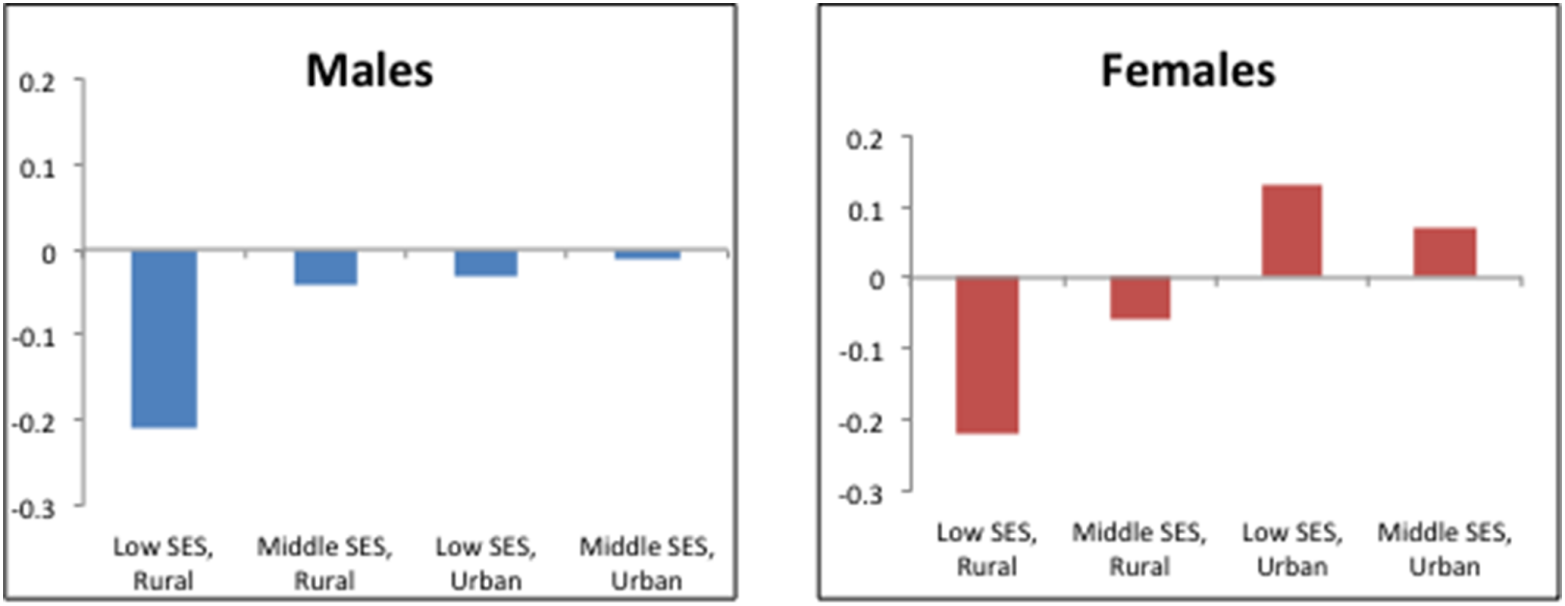
Association between SES and adherence to cancer prevention guidelines (β), stratified by gender and regional setting, 2002–2004 WHS.

There was a significant interaction between rural/urban residence and household SES (Figure 3). For men, regardless of regional setting, residing in low or middle SES households was associated with a reduction in adherence score compared with those in high SES households, although only the declines in rural settings were statistically significant (–0.27 and –0.05 for low and middle SES compared with high SES respectively). For women, residing in low and middle SES households compared with high SES households in rural regions was significantly associated with lower adherence scores (–0.28 and –0.10 for low and middle SES compared with high SES respectively). However, women in low and middle SES households compared with high SES households in urban regions had significantly higher adherence scores (0.09 and 0.05 for low and middle SES compared with high SES respectively).

## Discussion

The purpose of this analysis was to examine the level and predictors of adherence to cancer prevention guidelines among adults in 18 sub-Saharan African countries using the WCRF/AICR guidelines. Overall adherence to guidelines varied significantly between countries, and overall adherence score was less than 3.5 out of 5. Adherence was low for physical activity, nutrition, and BMI, but high for smoking and alcohol use. There was significant variation in adherence to specific guidelines between countries, and within countries there was significant variation between groups stratified by age, gender, household SES, overall health, and regional settings.

Adherence to the guidelines was highest among the young, women, high SES households, individuals in good overall health, and in those in urban areas. Individuals with these characteristics are likely to have resources to afford healthy diets, and educated enough to understand the risks associated with risky behavior such as smoking and excessive alcohol consumption. Our observation that adherence is higher among higher SES households is consistent with the fundamental causes of health theory suggesting that just as the adoption of new health behaviors start within higher socio-economic groups, reductions in those same behaviors also begin in those groups as information about the detrimental effects of such behaviors disseminate [29], [30]. For instance, in newly emerging economies, targeted marketing of high fat and high calorie diets or tobacco products would focus on high SES adults who are able to afford such products, increasing consumption in this group. However, due to better access to medical care in high SES groups compared with other groups, high SES groups are also more likely to receive timely information on the detrimental health effects of the products, making them more likely to eventually reduce consumption of such products. However, detailed exploration of these trends is beyond the scope of this paper. A future longitudinal study assessing changes in risk factors in SES groups over time during economic transition would be an ideal study design to explore these issues further.

We also observed an interaction on the additive scale between household SES and regional setting on adherence to cancer prevention guidelines. The interaction may be due to regional differences in the impact of income, since durable household goods were used in the development of the household SES construct. In rural areas, belonging to a high SES household may mean the possession of land for farming, tools for more efficient use of farmland, and the possession of livestock. However, in urban areas, a high SES household may be defined instead by the possession of a car, TV in the house, and a cell phone. For the nutrition, alcohol and tobacco components of the cancer adherence guidelines, these regional differences may have a significant impact. In urban areas, residing in a high SES household would imply more exposure to advertisement for high fat foods, cigarette and alcohol. While in rural areas, residing in a high SES household would imply access to fresh, healthy food. Efforts to promote health education and awareness of cancer prevention would therefore have to take regional differences into account, and take advantage of the existing communication infrastructure in urban areas to reach high SES women who may be more exposed to unhealthy TV advertising.

Although a prior study has shown a relationship between increased adherence to the WCRF/AICR guidelines and decreased mortality [21], few studies have examined guideline adherence or identified predictors associated with guideline adherence within developing regions. One study examining guideline adherence in Netherlands, Scotland, Mexico, and Guatemala, showed that Guatemala reached 10 of the 12 nutrition guidelines while Mexico only reached 7 of the 12 [31]. While there are limited studies that use WCRF/AICR adherence scores at the population level in other developing regions, the current literature provides support of our findings that adherence to cancer prevention risk factors is low within certain population groups in many developing regions [32], [33].

Our findings suggest that in addition to demographic and socioeconomic factors, the proportion of a country’s GDP spent on health also significantly improved adherence for both genders, although the effect was stronger on women. Other studies have reported on the significant impact of increased health spending on health status within countries [27], [34]. A recent study on the impact of government health expenditure on infant and childhood mortality reported a 13% and 32% reduction when the share of GDP spent on health is doubled [34]. Most of the countries in the present analysis spent between 3% and 8% of their GDP on health in 2005. The low levels of health spending suggest that there is significant room for improvements in adherence if efficient investments are made in the health system.

Studies on diet and physical activity in the US and parts of Europe also report low levels of adherence to cancer prevention guidelines. A 2009 report indicated that only 23% of adults in the US consumed 5 or more servings of fruits and vegetables per day [35] and in England only about 30% of adults consumed 5 or more servings of fruits and vegetables per day [36]. Although prevalence of smoking and alcohol was generally low in this study, there were large between-country and gender differences, with men still smoking at higher rates than women in all the countries. Current smoking rates ranged from 40% among men in South Africa, to under 8% in Ethiopia. In comparison, about 20% of adult men in the US reported currently smoking cigarettes in 2009, and 23% of adult men were current smokers in England in 2011 [35], [36]. Clearly, all countries need to devote significant efforts to improve adherence to cancer prevention guidelines. In countries where cultural or religious norms exert a positive influence on cancer risk factors, such norms need to be supported and encouraged. For instance adherence to alcohol guidelines are close to 100% in several Islamic African countries such as Senegal, Mali, Comoros, and Mauritania.

There are several strengths and limitations of our study. This is one of very few analyses examining and documenting the impact of modifiable lifestyle risk factors relevant to cancer risk in African populations. Data was obtained from a standardized multi-country survey with a focus on multiple items relating to health. Therefore, while we cannot rule out socially desirable answers on any one part of the survey such as reported alcohol use in Islamic countries, it is not likely to be a major source of measurement error. In addition, household SES was created using a composite set of variables relating to durable household goods. This approach is commonly used in research assessing SES in low-income areas, since household income and education do not adequately capture SES in areas where durable goods are more meaningful [25]. The WCRF/AICR scores used in this study assumed equal weights of each of the component risk factors. However, this is a common assumption that is valid for examining the prevalence of a collection of risk factors within populations, and for assessing disease outcomes comparing adherent and non-adherent adults [37]–[39]. Finally, the WHS was a cross-sectional study designed to assess health status in multiple countries at one time-point. Therefore, results presented here do not directly imply a causal association between individual or country level factors and adherence to cancer prevention guidelines. Future studies, including prospective studies, are needed to understand the impact of these risk factors and how changes in the prevalence of risk factors influence cancer risk.

In conclusion, there were significant between- and within-country differences in levels of adherence to current cancer prevention guidelines in Africa. The current analysis was based on the most current WHS data from 2002–2004. If observed patterns of low adherence level persist, as is expected given the rapid westernization of lifestyle experienced in many African countries, cancer incidence can be expected to increase dramatically in coming decades. These results suggest that significant amounts of resources and an integrated government response are needed to increase adherence to these guidelines, especially in the least adherent population sub-groups. Adherence to the WCRF guidelines will help ensure that cancer rates, already increasing rapidly in these regions, will stabilize in the coming decades.

## Supporting Information

File S1 (DOCX)
