# Supplementary material for: Adherence to Cancer Prevention Guidelines in 18 African Countries
Source: PLoS One. 2014 Aug 21;9(8):e105209. doi: 10.1371/journal.pone.0105209 (PMC4140739; doi:10.1371/journal.pone.0105209)
Supplement: File S1 — (DOCX) [file pone.0105209.s001.docx]

Table 1: Age-adjusted cancer incidence per 100,000 and data source for 18 sub-Saharan African countries, GLOBOCAN 2008

| **Country** | **WHS Sample Size** | **Female Breast** | **Cervix** | **Colorectal** | **Liver** | **Prostate** | **Data Source** |
| --- | --- | --- | --- | --- | --- | --- | --- |
| **Africa Region** |  | 25.2 | 28 | 5.9 | 8.3 | 17.5 | Cancer Registry Data Information |
| Cote D'Ivoire | 3241 | 22.7 | 26.9 | 3 | 6.7 | 34.8 | Incidence rates for the country have been estimated as the incidence rates from Abidjan Cancer Registry (1995-1998) and applied to the 2008 population. |
| Congo (Republic) | 3068 | 26.3 | 27.2 | 4.9 | 14.7 | 31 | Incidence rates for the country have been estimated as the Brazzaville incidence rates 1998-20021 and applied to the 2008 population |
| Comoros | 1835 | 12.3 | 51.7 | 2.4 | 4.3 | 12.5 | No data. Incidence rates for the country have been estimated as the weighted average of estimates for Malawi, Tanzania and Zimbabwe. |
| Ethiopia | 4957 | 19.5 | 18.8 | 6 | 5.2 | 6.3 | No data. Weighted average of estimates for Sudan and Kenya |
| Ghana | 4158 | 25.8 | 29.5 | 3.8 | 17.4 | 20.4 | No data. Incidence rates for the country have been estimated as the simple mean of estimate for Cote d’Ivoire, Niger and Burkina Faso. |
| Kenya | 4353 | 23.9 | 23.4 | 4.8 | 6.6 | 15.2 | Incidence rates for the country have been estimated as the simple mean of the rates from: Eldoret Cancer Registry (1998-2002) |
| Mali | 5168 | 23.8 | 37.7 | 5.8 | 13.7 | 13.1 | Incidence rates for the country have been estimated as the incidence rates from the Bamako cancer registry (2000-2006) and applied to the 2008 population. |
| Mauritania | 3836 | 23.9 | 35.1 | 4.4 | 16.4 | 17.9 | No data. Incidence rates for the country have been estimated as the unweighted average of Cote d’Ivoire, Abidjan (1995-1998), Guinea, Conakry (2000-2005), Mali, Bamako (2000-2006), Niger, Niamey (2001-2007), Nigeria, Ibadan (1999-2001) and The Gambia (1997-1998) cancer registries. |
| Malawi | 5541 | 12.1 | 50.8 | 2.6 | 4.3 | 13.5 | Incidence rates for the country have been estimated as the incidence rates for Blantyre districts (urban and rural) 1998-20041 applied to the 2008 population. |
| Senegal | 3249 | 23.7 | 34.7 | 4.3 | 16.5 | 18.1 | Incidence rates for the country have been estimated as the unweighted average of Cote d’Ivoire, Abidjan (1995-1998), Guinea, Conakry (2000-2005), Mali, Bamako (2000-2006), Niger, Niamey (2001-2007), Nigeria, Ibadan (1999-2001) and The Gambia (1997-1998) cancer registries. |
| Swaziland | 3064 | 9.88 | 50 | 2.2 | 9.8 | 17.6 | Incidence rates from the national cancer registry (1998-2002) applied to 2008 population |
| Chad | 4864 | 30.6 | 19.9 | 5 | 8.6 | 14 | Incidence rates for the country have been estimated as the simple mean of estimates for Sudan, Niger, Nigeria and Cameroon. |
| South Africa | 2629 | 41 | 26.6 | 14.5 | 9.5 | 59.7 | South Africa Cancer Registry incidence rates (1995-2001, pathological based series), were projected to 2008 and “scaled” by cancer, sex and age-specific percentages of microscopically verified cases observed in Zimbabwe, Harare City cancer registry (source CI5 Vol. IX). |
| Zambia | 4164 | 20.5 | 52.8 | 4.7 | 4.2 | 30 | No data available. Incidence rates for the country have been estimated as the simple mean of the rates from: Eastern Africa estimated rates. |
| Zimbabwe | 4252 | 16.2 | 47.4 | 5.4 | 6.9 | 26 | Incidence rates for the country have been estimated as the simple mean of the rates from: Harare Cancer Registry (2000-2005), black population |

Table 2: Proportion of Adults Adherent to Smoking, Alcohol, BMI, Physical Activity and Nutrition Guidelines by Country, WHS 2002-2004

|  | **Physical Activity** | | **Smoking** | | **Alcohol** | | **BMI** | | **Nutrition** | |
| --- | --- | --- | --- | --- | --- | --- | --- | --- | --- | --- |
| **Country** | Women | Men | Women | Men | Women | Men | Women | Men | Women | Men |
| Mauritius | 3.4 | 5.3 | 82.7 | 72.1 | 85.5 | 87.3 | 1.8 | 9.2 | 1.4 | 2.6 |
| Burkina Faso | 8.3 | 8.5 | 87.0 | 61.0 | 92.1 | 86.4 | 7.3 | 24.9 | 10.8 | 10.6 |
| Zimbabwe | 11.8 | 12.8 | 87.3 | 71.0 | 94.8 | 93.9 | 18.9 | 18.9 | 10.9 | 11.5 |
| Malawi | 11.9 | 12.3 | 88.3 | 75.2 | 95.1 | 88.7 | 20.1 | 23.3 | 12.9 | 12.3 |
| Namibia | 14.9 | 19.7 | 94.2 | 74.2 | 97.4 | 93.8 | 20.6 | 24.6 | 13.5 | 14.3 |
| Mali | 17.5 | 16.2 | 94.7 | 76.0 | 97.6 | 96.4 | 22.6 | 29.2 | 14.2 | 13.1 |
| Kenya | 20.2 | 23.7 | 95.6 | 76.3 | 98.0 | 96.4 | 26.3 | 41.3 | 15.5 | 18.2 |
| Cote D'Ivoire | 20.3 | 23.7 | 95.7 | 73.4 | 98.1 | 92.7 | 31.5 | 41.1 | 15.9 | 18.3 |
| Mauritania | 21.9 | 32.4 | 95.9 | 84.5 | 98.8 | 97.9 | 37.4 | 41.4 | 19.3 | 22.3 |
| Ghana | 22.8 | 22.9 | 96.5 | 80.8 | 99.3 | 94.9 | 39.4 | 49.5 | 23.2 | 21.3 |
| Swaziland | 25.4 | 27.0 | 96.8 | 72.8 | 99.3 | 95.0 | 40.9 | 44.6 | 25.3 | 22.0 |
| Congo | 27.6 | 31.2 | 96.9 | 79.3 | 99.3 | 98.2 | 48.3 | 52.9 | 26.6 | 25.3 |
| Ethiopia | 28.3 | 33.4 | 97.1 | 57.3 | 99.7 | 99.9 | 51.0 | 58.6 | 27.9 | 31.2 |
| Senegal | 30.7 | 31.7 | 98.1 | 76.6 | 99.8 | 97.1 | 55.8 | 66.3 | 28.1 | 32.7 |
| South Africa | 49.8 | 52.6 | 98.2 | 73.0 | 99.8 | 93.7 | 56.9 | 60.1 | 28.3 | 30.6 |
| Comoros | 66.7 | 70.5 | 98.2 | 83.5 | 99.9 | 99.3 | 61.1 | 63.2 | 32.5 | 28.5 |
| Chad | 68.7 | 71.0 | 98.5 | 88.9 | 100 | 99.9 | 61.2 | 57.9 | 58.6 | 62.1 |
| Zambia | 80.6 | 83.8 | 99.4 | 92.7 | 100 | 100 | 61.5 | 72.6 | 61.3 | 60.5 |
